# Familial and Clinical Correlates in Depressed Adolescents with Borderline Personality Disorder Traits

**DOI:** 10.3389/fped.2016.00087

**Published:** 2016-09-21

**Authors:** Jean-Marc Guilé, Christophe Huynh, Jean-Jacques Breton, Sébastien Garny De La Rivière, Claude Berthiaume, Marie St-Georges, Réal Labelle

**Affiliations:** ^1^Research Department, Fernand-Seguin Research Centre, Rivière-des-Prairies Hospital, Montreal, QC, Canada; ^2^Université Picardie Jules Verne, Amiens, France; ^3^McGill University, Montreal, QC, Canada; ^4^Department of Psychiatry, Faculty of Medicine, Université de Montréal, Montreal, QC, Canada; ^5^Centre for Research and Intervention on Suicide and Euthanasia, Université du Québec à Montréal, Montreal, QC, Canada

**Keywords:** borderline personality disorder, depressive disorder, adolescent, chart review, aggression, family, correlates, psychopathology

## Abstract

**Introduction:**

Chart review is a low-cost, but highly informative, method to describe symptoms, treatment, and risk factors associated with borderline personality disorder (BPD) and to adapt screening and intervention to clinical reality. Previous chart review studies report more aggressiveness/anger and psychotic features in youths with BPD. They show that adverse family environment and parental psychopathology constitute important factors for BPD pathology.

**Objectives:**

To examine clinical characteristics of depressed BPD adolescents (12–17 years old) outpatients according to gender and to explore variables which are associated with BPD traits.

**Methods:**

A retrospective chart review using the child and adolescent version of the retrospective diagnostic instrument for borderlines was conducted on 30 depressed adolescents with BPD traits and 28 depressed patients without BPD traits. Participants who reached the retrospective diagnostic instrument for borderlines threshold for BPD were included in the BPD traits group. Comparison analyses were performed using Pearson’s Chi-square test. Associated factors were determined using regression analyses.

**Results:**

BPD traits participants were characterized by higher family problems (parental psychopathology, parent disagreement/argument, and parent–child relational problem), more aggressive symptoms, and higher rates of family intervention and hospitalization. A number of familial factors (parental history of delinquency, substance use, personality disorders, having siblings, or parental disagreement/argument in boys) were associated with BPD traits. Attention seeking and problematic functioning (does not adapt well to group activities) were also associated with BPD traits.

**Discussion:**

Our study stresses the need to assess BPD traits in adolescent psychiatric evaluation, especially in the presence of aggressive behaviors, family problems and attention seeking. Our results also highlight the importance of exploring family characteristics intervention in adolescents with BPD traits.

## Introduction

Considering its high association with suicidal behaviors and its alarming prevalence in the general adolescent population (15–20%), it is surprising to find few descriptive studies on depressive disorders (DD) in clinical youth population (1). Major DD is the main contributor for suicide whereas borderline personality disorder (BPD) is the greatest source of suicidal behaviors in adolescents. The overlap between DD and BPD is commonly noticed in clinical settings receiving adolescents. Compared with adolescents without BPD, adolescents with BPD present more severe suicidal ideation and behaviors, and a higher score for depression as assessed with the BDI-II self-report (2) as well as a higher frequency of DD as evaluated by the NIMH-DISC IV (3).

With respect to the DSM-5 classification, BPD is characterized by affective instability, intense and unstable relationships, marked impulsivity, distorted cognitions, suicidal behaviors, and non-suicidal self-injury (4, 5). During adolescence, BPD affects 53% of inpatients, 11% of outpatients, and 3% of adolescents in a community sample (2). Studies on children and adolescents with BPD are still rare since many psychiatrists are reluctant to diagnose this disorder in youths, despite growing consensus on the construct (6, 7). The belief that personality disorders only exist in adults still remains popular, though some recognize that they do not appear *ex nihilo* in adulthood. In fact, as previous versions, the fifth edition of the DSM clearly states that BPD can be diagnosed before the age of 18 if the youth’s maladaptive personality traits appear to be persistent and unlikely to be restricted to a particular developmental stage (4, 5). Thus, using DSM criteria, prevalence of BPD varies between 0.9 and 3% for adolescents (8–10). Clinical and structured interviews and self-reported questionnaires can reliably identify adolescents with BPD symptomatology deviating strongly from typical adolescence (11–14). Longitudinal studies have demonstrated that BPD is relatively stable during adolescence, with a moderate decline onward (15, 16). Retrospective studies also show that BPD adults reported onset of symptoms such as strong emotional reactivity and low frustration tolerance during childhood (17). Evolution is less favorable for BPD adolescents: life satisfaction, social support, relationship quality, integration into adult life, and education level are lower for these patients compared to adolescents who did not have this diagnosis (18). Therefore, previous works have shown that BPD can be reliably diagnosed in children and adolescents using a valid construct similar to adult BPD (6). Early diagnosis nowadays is encouraged (19) and some countries, like Australia, have issued practice guidelines to promote diagnostic and therapeutic services dedicated to youths with BPD symptomatology (20).

A limited number of retrospective chart reviews investigating adolescent BPD as the main subject of interest have been published in the last 25 years. Chart review research implies analyzing data that have been originally collected for reasons other than research, including all available clinical data included in medical files (21). James et al. (22) reported higher rates of intense affects, interpersonal psychopathology (manipulation, devaluation, and boredom), sexual abuse, and maternal helplessness in 24 BPD adolescents (14 out of which had MDD). Diagnosis was made using the diagnostic interview for borderlines (DIB). They were usually referred for overdose and major DD. They tended to regress during hospitalization and had intense, but short, episodes of anger and depression. The authors also observed a high rate of wrist cutting in response to intolerable tension, suicide attempts, and absconding. Family environment was described as being more volatile, disturbed, and filled with anger (22).

Another chart review study emphasized the etiological role of cumulative family factors in the development of 27 BPD female adolescent inpatients (DSM-III Axis I diagnoses were not reported). The authors reported on a number of variables: neglect, maternal rejection, grossly inappropriate parental behavior, parental loss, number of surrogate mothers and fathers, number of relocation, physical abuse, and sexual abuse, which were associated with BPD in adolescents (23).

Another chart study examined if BPD in adolescence and adulthood was similar in terms of symptoms and treatment. Compared to adults, BPD adolescents had higher rates of current non-suicidal self-injury and obsessive–compulsive symptoms, and past running away. Adults had more alcohol abuse than adolescents. A positive correlation was reported between the number of prescribed antipsychotics and the length of hospitalization. Apart from these characteristics, the study showed that symptoms and treatment between the two age groups were highly similar; this suggested that the diagnostic criteria of BPD have similar validity between adolescents and adults (24). Thus, BPD adolescents presented high rates of intense negative affects, relational problems, and self-injurious behaviors. Adverse familial environment, parental psychopathology, and cumulative abuse also constituted important risk factors for BPD pathology.

The aforementioned chart reviews furthered our knowledge either about adolescent depression or BPD in terms of clinical characteristics and risk factors. However, few descriptive studies have examined specifically adolescent DD with comorbid BPD. Our study aims to examine, by chart review, these clinical characteristics and risk factors in BPD depressed adolescents. Clinical differences between depressed BPD and non-BPD adolescents as well as gender differences will be explored. Answering these questions allows us to adapt screening and intervention according to the characteristics and needs for youths assessed and treated in child psychiatry.

## Materials and Methods

### Participants

Data were obtained through a retrospective chart review, which is a validated cost-effective means to obtain relevant sociodemographic and clinical information on children and adolescents treated in psychiatry (21). We examined medical files from all patients (*n* = 405) treated during the 2002–2003 school year at Rivière-des-Prairies Hospital, an out- and inpatient psychiatric hospital for children and adolescents affiliated with the University of Montréal. To be included in this study, patients had to be between 12 and 17 years old and treated for DD, following a standardized diagnostic evaluation performed by a mutidisciplinay team including a child psychiatrist according to the best estimate team consensus method (25). Procedure was reported in our previous study (1).

### Procedure

In accordance with ethics laws enforced in the province of Québec, authorization to access medical files was obtained from the Hospital’s Director of Professional Services. Two trained research assistants proceeded with the chart review, using a chart review instrument developed by two of the authors (Jean-Jacques Breton and Réal Labelle) to insure standardization of data collection (1). Prior to the data collection, they completed independent ratings on 10 files (interrater reliability: κ = 0.80). Data pertaining to sociodemographic characteristics, family history, personal history, DSM diagnoses, symptoms, and treatment were extracted using the instrument. Psychiatric diagnoses were recorded by the clinicians in the patient’s chart according to DSM-IV-TR criteria and categories [see previous publication: (1)]. In the current paper, psychiatric diagnoses were presented with respect to DSM-5 criteria and categories. Consequently, major DD and disruptive behavior disorder have been changed for DD and disruptive disorder and/or attention-deficit/hyperactivity disorder (ADHD), respectively. Changes between DSM-IV-TR and DSM-5 were as follows (4, 5): mood disorders have been split in two separate categories, bipolar disorders and DD, the latter including dysthymia (which is defined as a sub-type of persistent DD) and Major DD. «Neither the core criterion symptoms applied to the diagnosis of major depressive episode nor the requisite duration of at least 2 weeks has changed from DSM-IV. Criterion A for a major depressive episode in DSM-5 is identical to that of DSM-IV» (4, 5). As none of the participants presented bipolar disorders, the DSM-5 category of DD eventually appeared more appropriate than the DSM-IV-TR category of mood disorders. ADHD previously included in the DSM-IV category of disruptive behavior disorders has been replaced in the DSM-5 neurodevelopmental disorders section. Conduct disorder (CD) and oppositional defiant disorder (ODD) remained in the DSM-5 category of disruptive disorders. However, diagnostic criteria for ADHD and CD in DSM-5 are similar to those in DSM-IV-TR. Severity level and frequency of ODD symptoms have been increased in order to better discriminate with normal development. Anxiety disorders criteria, apart from obsessive–compulsive disorders, remained mostly unchanged.

To determine the presence (or absence) of BPD traits, the child and adolescent version of the retrospective diagnostic instrument for borderlines (C-DIB) was used (26). This instrument was adapted from the semi-structured interview DIB revised (27) to be used for retrospective chart review with child and adolescent clinical population. BPD traits are assessed through 24 items pertaining to social adaptation, impulsivity, affects, psychosis, and interpersonal relations. The maximum total scaled score is 10. A score >6 supports a diagnosis of BPD (28). Participants who reached the C-DIB threshold for BPD were included in the BPD traits group. Concerning internal consistency, a previous study conducted by one of the authors (Jean-Marc Guilé) reported the following Cronbach’s alpha coefficients for each section of this instrument: 0.24 (social adaptation), 0.74 (impulsivity), 0.39 (affective disturbances), 0.93 (psychotic features), 0.55 (relationship impairments), and 0.63 (total score). Section–total correlations were between 0.11 (lowest for psychosis section) and 0.62 (highest for relationship impairments section). The interrater reliability was established as good (κ = 0.72) (29).

### Data Analysis

All analyses were done using the statistical package for social sciences (SPSS). Missing values were imputed through statistical strategies as recommended in chart review research (21). We performed a univariate analysis to compare groups using Pearson’s χ^2^ test. A first comparison was conducted according to diagnosis (BPD traits vs. non-BPD traits). Then, we compared boys vs. girls in the BPD traits group only. If the expected cell count was less than five, we used Fisher’s exact test (two-sided).

We also performed a multivariate logistic regression analysis for independent variables that reached statistical significance in the comparison test. We defined the diagnostic traits group as the dependant variable. To determine which variables were associated with BPD traits, a multivariate linear regression was performed using the total C-DIB score as the dependant variable. Symptoms, which were part of BPD definition, such as aggressive behaviors, were removed from analyses. The level of significance was fixed at 0.05. We did not perform statistical correction because of the exploratory nature of our study and the small sample size.

## Results

DSM-5 diagnoses for the 58 patients who fit all the inclusion criteria for this study are presented in Table 1. All adolescents were diagnosed with a DD. None of them presented a bipolar disorder. With respect to comorbidity, the BPD traits group had more comorbid disruptive behavior disorders than the non-BPD traits group. In both groups, approximately one-fifth also presented an anxiety disorder. Familial problems were more frequent in the BPD traits group. For both groups, a high percentage of patients had moderate to serious global functional disruption. In the BPD traits group, pathological personality traits (among the traits for all DSM-5 personality disorders) were clinically confirmed for only 2 boys (14%). This result is in sharp contrast with the finding that 51.7% of our sample present BPD traits according to the C-DIB. For DSM-5 diagnoses, no significant difference was found between BPD boys and girls.

**Table 1:** **DSM-5 diagnoses and associated conditions**.

	BPD traits group			Non-BPD traits group
	Boys	Girls	Total	Total
**Psychiatric diagnoses**	***n* = 16**	***n* = 14**	***n* = 30**	***n* = 28**
Depressive disorder (major depressive disorder/dysthymia)	8 (50%)	6 (43%)	14 (48%)	17 (61%)
Depressive disorder with disruptive disorder and/or attention-deficit hyperactivity disorder	6 (38%)	4 (29%)	10 (33%)	5 (18%)
Depressive disorder with anxiety disorder	2 (13%)	4 (29%)	6 (20%)	6 (21%)
Intellectual disability	0 (0%)	1 (7%)	1 (4%)	2 (8%)
Personality disorder traits^a^	2 (14%)	0 (0%)	2 (7%)	3 (11%)
**Medical condition**	***n* = 14**	***n* = 14**	***n* = 28**	***n* = 28**
Present	2 (14%)	3 (21%)	5 (18%)	8 (29%)
**Social condition**	***n* = 16**	***n* = 14**	***n* = 30**	***n* = 28**
Familial problems	11 (69%)	10 (71%)	21 (70%)	18 (64%)
School problems	0 (0%)	1 (7%)	1 (3%)	3 (11%)
Other problems	3 (19%)	0 (0%)	3 (10%)	2 (7%)
**Global Assessment Functioning**	***n* = 14**	***n* = 14**	***n* = 28**	***n* = 28**
0–60 (moderate–serious)	11 (79%)	11 (79%)	22 (79%)	19 (68%)
61–100 (absent–mild)	3 (21%)	3 (21%)	6 (21%)	9 (32%)

*^a^All personality disorders traits were assessed by a clinician, not exclusively BPD*.

Family variables are presented in Table 2. In both groups, a high percentage of depressed adolescents lived in a family structure which was either reconstituted or monoparental. Significantly more non-BPD patients were single child than BPD patients (*p* = 0.0048). Concerning family history, initial analyses using Pearson’s Chi-Square showed a significant difference between BPD and non-BPD patients. In the BPD traits group, there was a significant higher frequency of delinquency/drug abuse/personality disorders in fathers of BPD patients (*p* = 0.029) compared to non-BPD adolescents. For the same problems, BPD patients’ mothers had also more problems related to delinquency/drug abuse/personality disorders (*p* = 0.055) than non-BPD adolescents’ mothers (non-significant trend). However, since two cells did not have the minimum expected count, we reran the analysis using Fisher’s exact test (two-sided). The results were no longer significant for fathers’ (*p* = 0.053) and mothers’ (*p* = 0.104) psychopathologies. For family structure, number of siblings, and family history, no statistical difference was found between BPD boys and BPD girls.

**Table 2:** **Family variables**.

	BPD traits group			Non-BPD traits group
	Boys *n* = 16	Girls *n* = 14	Total *n* = 30	Total *n* = 28
**Family structure**				
Biparental	5 (31%)	7 (50%)	12 (40%)	12 (43%)
Monoparental	4 (25%)	5 (36%)	9 (30%)	11 (39%)
Reconstituted	7 (44%)	2 (14%)	9 (30%)	5 (18%)
**Siblings^a^**				
Single child	0 (0%)	1 (7%)	1 (3%)	9 (32%)
Other siblings	16 (100%)	13 (93%)	29 (97%)	19 (68%)
**Family history**				
Mood disorder(s) – mother	4 (25%)	5 (36%)	9 (30%)	11 (39%)
Mood disorder(s) – father	0 (0%)	2 (14%)	2 (7%)	1 (4%)
Delinquency/drug abuse/personality disorder – mother	2 (13%)	4 (29%)	6 (20%)	1 (4%)
Delinquency/drug abuse/personality disorder – father	3 (19%)	4 (29%)	7 (23%)	1 (4%)

*^a^BPD total vs. non-BPD total: p ≤ 0.01*.

Table 3 presents past life events and symptomatic traits observed or reported during the initial psychiatric evaluation. In the BPD traits group, more adolescent boys witnessed parent disagreement and argument than girls (*p* = 0.011). As a group, this variable was more present in BPD patients than non-BPD patients (*p* = 0.009).

**Table 3:** **Most frequent life events and clinical symptoms according to gender and diagnosis**.

	BPD traits group			Non-BPD traits group
	Boys *n* = 16	Girls *n* = 14	Total *n* = 30	Total *n* = 28
**Life events**				
Parent divorce/separation	11 (68%)	6 (43%)	17 (57%)	15 (54%)
Parent disagreement/argument^a^	**13 (81%)**	**5 (36%)**	**18 (60%)**	**9 (32%)**
Physical violence/abuse and sexual abuse/rape	8 (50%)	5 (36%)	13 (43%)	8 (29%)
Foster placement	3 (19%)	4 (29%)	7 (23%)	4 (14%)
**BPD traits**				
Sadness	12 (75%)	14 (100%)	29 (87%)	22 (79%)
Suicidal ideation	13 (81%)	12 (86%)	25 (83%)	21 (75%)
Oppositional, sarcastic, and hostile^b^	13 (81%)	12 (86%)	**25 (83%)**	**5 (18%)**
Does not adapt well to group activities	8 (50%)	8 (57%)	16 (53%)	9 (32%)
Loss of interest^c^	**8 (50%)**	**13 (93%)**	21 (70%)	21 (75%)
Sleep problems	8 (50%)	12 (86%)	20 (67%)	17 (61%)
Low self-esteem	12 (75%)	9 (64%)	21 (70%)	16 (57%)
Does not fulfill his/her potential at school	10 (63%)	10 (71%)	20 (67%)	12 (43%)
Isolated, few friends	8 (50%)	7 (50%)	15 (50%)	9 (32%)
**Other features**				
Parent–child relational problem^d^	15 (94%)	14 (100%)	**29 (97%)**	**21 (75%)**
School achievement difficulties	12 (75%)	9 (64%)	21 (70%)	13 (46%)
Concentration problems	12 (75%)	8 (57%)	20 (67%)	16 (57%)
Insecurity	10 (63%)	7 (50%)	17 (57%)	11 (39%)
Attention seeking^e^	11 (69%)	10 (71%)	**21 (70%)**	**9 (32%)**
Difficult to control^f^	14 (88%)	8 (57%)	**22 (73%)**	**6 (21%)**
Fit of rage^g^	13 (81%)	9 (64%)	**22 (73%)**	**9 (32%)**
Feeling devalued	9 (56%)	10 (71%)	19 (63%)	15 (54%)
Anxiety	10 (63%)	12 (86%)	22 (73%)	19 (68%)
Aggression toward parents^h^	11 (69%)	11 (79%)	**22 (73%)**	**6 (21%)**
Being threatened, insulted, or assaulted by others	4 (25%)	4 (29%)	8 (27%)	5 (18%)
Threatening, calling names, or insulting others^i^	4 (25%)	5 (36%)	**9 (30%)**	**0 (0%)**
Assaulting others^j^	6 (38%)	5 (36%)	**11 (37%)**	**1 (4%)**

*Significant differences are highlighted in bold*.

*^a^BPD boys vs. BPD girls: p = 0.011; BPD total vs. non-BPD total: p = 0.009*.

*^b^BPD total vs. non-BPD total: p ≤ 0.001*.

*^c^BPD boys vs. BPD girls: p = 0.017*.

*^d^BPD total vs. non-BPD total: p = 0.023*.

*^e^BPD total vs. non-BPD total: p = 0.004*.

*^f^BPD total vs. non-BPD total: p ≤ 0.001*.

*^g^BPD total vs. non-BPD total: p = 0.002*.

*^h^BPD total vs. non-BPD total: p ≤ 0.001*.

*^i^BPD total vs. non-BPD total: p = 0.002*.

*^j^BPD total vs. non-BPD total: p = 0.002*.

Parent–child relational problem was high in both groups (BPD and non-BPD), but it was significantly higher in the BPD traits group (*p* = 0.023). Relational problems went as far as aggression toward parents, which was found in a significantly higher proportion in the BPD traits group (*p* ≤ 0.001). BPD adolescents presented a wider range of disruptive behaviors, from oppositional, sarcastic, and hostile attitude (*p* ≤ 0.001) to violent behaviors toward others such as threatening, calling names, insulting (*p* = 0.002), fit of rage (*p* = 0.002), and assaulting (*p* = 0.002). They were more attention seeking (*p* = 0.004) and more difficulty to control (*p* ≤ 0.001). Compared to BPD boys, BPD girls presented more loss of interest (*p* = 0.017) and more sleep problems (non-significant trend; *p* = 0.058). Boys and girls did not distinguish from each other in the other symptoms.

Data concerning treatment are presented in Table 4. Family intervention was more often present as being part of the treatment in the BPD traits group (52%) compared to the non-BPD (16%; *p* = 0.007). A higher frequency of BPD patients (45%), compared to non-BPD (4%), was hospitalized (*p* = 0.001). Medication was prescribed in a similar proportion between the two groups. However, in the BPD traits group, girls received more antidepressants or mood stabilizers while boys received other classes of medication (*p* = 0.024). A combination of psychotherapy and medication was the privileged treatment regimen.

**Table 4:** **Treatment**.

	BPD traits group			Non-BPD traits group
	Boys	Girls	Total	Total
	***n* = 15**	***n* = 11**	***n* = 26**	***n* = 24**
Psychotherapy	9 (60%)	10 (91%)	19 (73%)	14 (58%)
	***n* = 15**	***n* = 12**	***n* = 27**	***n* = 25**
Familial intervention^a^	6 (40%)	8 (67%)	14 (52%)	4 (16%)
	***n* = 14**	***n* = 14**	***n* = 28**	***n* = 25**
**Medication^b^**	9 (64%)	13 (93%)	22 (79%)	18 (72%)
Antidepressants or mood stabilizers	2 (14%)	7 (50%)	9 (32%)	11 (44%)
Antidepressants combined with other drug(s)	2 (14%)	5 (36%)	7 (25%)	5 (20%)
Other class of medication	5 (36%)	1 (7%)	6 (21%)	2 (8%)
	***n* = 15**	***n* = 14**	***n* = 29**	***n* = 25**
**Hospitalization^c^**	6 (40%)	7 (50%)	13 (45%)	1 (4%)
	***n* = 14**	***n* = 14**	***n* = 28**	***n* = 25**
Psychotherapy combined with medication	12 (86%)	14 (100%)	26 (93%)	22 (88%)
Treatment termination	***n* = 7**	***n* = 5**	***n* = 12**	***n* = 16**
Improvement	2 (29%)	2 (40%)	4 (33%)	2 (13%)
Lack of motivation	4 (57%)	3 (60%)	7 (58%)	11 (69%)
Transfer/relocation	1 (14%)	0 (0%)	1 (8%)	3 (19%)

*^a^BPD total vs. non-BPD total: p = 0.007*.

*^b^BPD boys vs. BPD girls: p = 0.024*.

*^c^BPD total vs. non-BPD total: p = 0.001*.

To determine which variables were associated with the presence of BPD traits at a score of 6 or higher according to the C-DIB (BPD traits group), we performed a multiple logistic regression (Table 5). Having siblings instead of being an only child (OR = 54.7; *p* = 0.002), having a parent (father or mother) with delinquency problems/drug abuse/personality disorders (OR = 25.0; *p* = 0.002), witnessing parental disagreement and argument (for boys only) (OR = 22.2; *p* = 0.004), and seeking attention (OR = 11.0; *p* = 0.002) were significantly associated with the BPD traits group.

**Table 5:** **Variables associated with the borderline personality disorder traits group in adolescents: multivariate logistic regression model (*n* = 58)**.

	Odds ratio	95% CI	*p*
Having siblings vs. single child	54.7	1.5–2021.0	0.002
Father’s or mother’s delinquency/substance use/personality disorder	25.0	1.7–363.8	0.002
Personal history of parent disagreement or argument *for boys*	22.2	1.6–298.8	0.004
Attention seeking	11.0	1.7–69.8	0.002

We determined which variables would be associated with the number of BPD traits using a multivariate linear regression model (Table 6). The final model explained 31.4% of the variance observed. Presence of parental disagreement or argument for boys, attention seeking, and difficulty to adapt to group activities were associated with the number of BPD traits. Considering the three independent variables, which were significantly associated with BPD traits and the sample size of 58 participants, *post hoc* power calculation yielded an estimated power of 0.987 for an alpha error fixed at 0.05.

**Table 6:** **Variables associated with BPD traits in adolescents: multivariate linear regression model (*n* = 58)**.

	*B*	95% CI	*p*
Parent disagreement or argument *for boys*	1.57	0.41–2.73	0.036
Attention seeking	0.98	0.21–1.75	0.014
Does not adapt well to group activities	0.84	0.07–1.62	0.033

*Final model adjusted R^2^ = 31.4*.

## Discussion

This chart review examined clinical characteristics of depressed adolescents with BPD traits. Two sets of comparisons were performed: (1) according to diagnosis (BPD traits vs. non-BPD traits groups), (2) according to gender for the BPD traits group. It also explored variables, which were associated with BPD traits.

Our study shows that a very small number of patients were given a personality disorder diagnosis by their treating psychiatrist. In contrast, using the C-DIB, we determined that half of the depressed adolescents had many BPD traits, which strongly suggest the presence of this diagnosis. Previous studies had shown the high prevalence of adolescent BPD in clinical settings. Thirty percent of depressed adolescent in- and outpatients met all the criteria for BPD (30). Among juvenile delinquents and hospitalized adolescents, BPD represented 31–62% of these populations (2, 3, 31). About 91% of adolescents seen at the emergency department of the McGill University Health Center in Montréal for a suicide attempt had BPD (32). Taken together, these results from different studies suggest that a high number of adolescent patients have BPD, but they rarely receive this diagnosis from a clinician. This stresses the importance to assess personality traits in clinical settings so the proper treatment can be given, as recommended by national guidelines, such as the Australian practice guideline (20).

Our results highlight the contribution of familial factors in BPD pathology. In our sample, compared to non-BPD depressed adolescents, a higher percentage depressed adolescents with BPD traits came from families where a parent, most often the father, had a personality disorder, a substance abuse, and/or delinquency problems. For boys, disagreement or argument among parents was associated with BPD traits. Parent–child relational problem was also more prevalent in the BPD traits group. In addition, depressed adolescents with BPD traits were more often having siblings, compared to non-BPD depressed adolescents. These findings are in agreement with previous clinical and population-based studies: a potentially harmful family environment with suboptimal parenting without necessary documented maltreatment occurrences can be a contributing factor for the development of BPD in adolescents (22, 23, 33). Previous reports show that paternal psychopathologies such as depression and substance use have a greater impact on the development of internalized and externalized symptoms in boys than in girls (34). One explanation is that boys seek more time with their father and view him as a strong role model. Based on our results, it is probable that boys with BPD traits may be more affected by the negative family environment if the father’s psychological difficulties contribute to it.

With respect to the clinical presentation, aggression distinguishes depressed adolescents with BPD traits from non-BPD depressed adolescents. This encompasses a wide range of behaviors: hostility and opposition, threats and insults toward others, physical assault, and aggression toward parents. Inappropriate anger or difficulty controlling anger constitutes one of the DSM-5 criteria needed for a BPD diagnosis (4, 5). A previous study showed that absence of anger or aggression was the best exclusion criterion for adolescent BPD (35). According to another report, BPD boys displayed more disruptive, aggressive, and antisocial behaviors than BPD girls (36). Though we have not observed this difference in our sample, Bradley’s study highlighted the presence of aggressive behaviors in BPD for boys. Thus, aggressive features seem to characterize adolescent BPD. This was supported by a recent study on hospitalized patients, which detailed highest comorbidities observed in BPD adolescents (3). ODD was the most frequently associated disorder in BPD adolescents in comparison to non-BPD inpatients (61.9 vs. 48.2% according to parent’s report). Our results are also consistent with the higher level of oppositional behaviors we previously observed in BPD suicidal adolescents in comparison with non-BPD suicidal adolescents (2). A high level of aggressive and oppositional behaviors in a depressed adolescent should be a signal for clinicians to assess BPD traits.

In our study, we found that girls with BPD traits reported more sleep problems and suffered more from loss of interest than boys. Though sleep is rarely studied in adolescent BPD, previous reports suggest the presence of sleep disruptions in this population (37, 38). Further investigation in this area is necessary since sleep quality has a role in affective regulation (39). In adult BPD, anhedonia is present during dysphoric states along with feelings of emptiness and boredom. According to the neurobiological model of BPD, loss of pleasure or interest, along with self-injurious behaviors, may be caused by an altered endogenous opioid system (40). Further research is needed to explain these sex differences and to determine if they have a biological basis.

Attention seeking was frequently found in the BPD traits group compared with the non-BPD traits group. This symptom was associated with a sufficient number of BPD traits to warrant a BPD diagnosis according to the C-DIB. Interpersonal relations are highly disturbed in this personality disorder. High attention seeking may be a way to avoid loneliness (frantic effort to avoid abandonment) and may explain partly why relationships are unstable and chaotic in these patients. Further investigations are needed on attention seeking and its role in BPD. Treating this symptom at an early age may reduce the development and crystallization of a full-blown BPD psychopathology.

The higher number of patients hospitalized in the BPD traits group compared with the non-BPD traits group underlines the complexity and the severity of depression when combined with a personality disorder. More patients with BPD traits had family intervention than non-BPD adolescents. This underscores the prominent role of disrupted familial environment in adolescent BPD. Addressing this issue through family intervention helps reducing the impact of these factors on everyday functioning. The importance of considering family issues and even more, offering family support, and counseling to BPD relatives, has been stressed by national guidelines, as the Australian practice guideline (20).

More antidepressants were prescribed to girls with BPD traits, while other classes of medication such as antipsychotic and stimulants were given to boys with BPD traits. This suggests that disruptive and aggressive behaviors were more often targeted in boys while girls were more often treated for their depressive and internalized symptoms. This result may reflect Bradley’s findings that BPD boys presented more externalized symptoms while BPD girls had an internalized profile (36).

Our results should be interpreted carefully since our study is not void of limitations. This being a retrospective chart review, some data have been missing, either because the information was not written in the file or the research assistants did not find it. Also, errors concerning data codification might have occurred. We only focused on depressed adolescents, so these findings cannot be generalized to other populations. A certain number of depressed adolescents might have evolved as bipolar disordered youths (41). Other comorbidities might have been overlooked. Using a retrospective questionnaire, the C-DIB, to determine the presence of BPD might have generated false-positives and false-negatives. Since BPD was not routinely assessed in child and adolescent psychiatry, we cannot determine if all the participants in the BPD traits group would have had this diagnosis if they were properly assessed for personality disorders during the initial psychiatric evaluation.

## Conclusion

This retrospective chart review underscores the need to assess personality disorders traits in adolescents. Presence of aggression, disruptive family environment (presence of parental psychopathology and intense conflicts among family members), and high attention seeking, all justify the need for a detailed assessment of BPD traits and family characteristics. Identifying BPD in its early stages allows for timely proper treatment and avoids complications related to misdiagnosing. It may also help reducing its symptoms, and its impact on individual and family functioning.

## Author Contributions

J-MG: design of the study, literature review, analyses and discussion, writing, and final revision. CH: literature review, data collection, analyses and discussion, and writing. SR: literature review, analyses and discussion, and writing. J-JB: design of the study, literature review, analyses and discussion, and writing. CB: analyses and discussion, writing. MS-G: data collection, analyses and discussion, and writing. RL: literature review, analyses and discussion, and writing.

## Conflict of Interest Statement

The authors declare that the research was conducted in the absence of any commercial or financial relationships that could be construed as a potential conflict of interest.
